# Dynamic Brains and the Changing Rules of Neuroplasticity: Implications for Learning and Recovery

**DOI:** 10.3389/fpsyg.2017.01657

**Published:** 2017-10-04

**Authors:** Patrice Voss, Maryse E. Thomas, J. Miguel Cisneros-Franco, Étienne de Villers-Sidani

**Affiliations:** Department of Neurology and Neurosurgery, Montreal Neurological Institute, McGill University, Montreal QC, Canada

**Keywords:** neuroscience, plasticity regulators, brain, plasticity, learning, recovery, regulators, neuromodulatory systems

## Abstract

A growing number of research publications have illustrated the remarkable ability of the brain to reorganize itself in response to various sensory experiences. A traditional view of this plastic nature of the brain is that it is predominantly limited to short epochs during early development. Although examples showing that neuroplasticity exists outside of these finite time-windows have existed for some time, it is only recently that we have started to develop a fuller understanding of the different regulators that modulate and underlie plasticity. In this article, we will provide several lines of evidence indicating that mechanisms of neuroplasticity are extremely variable across individuals and throughout the lifetime. This variability is attributable to several factors including inhibitory network function, neuromodulator systems, age, sex, brain disease, and psychological traits. We will also provide evidence of how neuroplasticity can be manipulated in both the healthy and diseased brain, including recent data in both young and aged rats demonstrating how plasticity within auditory cortex can be manipulated pharmacologically and by varying the quality of sensory inputs. We propose that a better understanding of the individual differences that exist within the various mechanisms that govern experience-dependent neuroplasticity will improve our ability to harness brain plasticity for the development of personalized translational strategies for learning and recovery following brain injury or disease.

## Introduction

Neuroplasticity can be viewed as a general umbrella term that refers to the brain’s ability to modify, change, and adapt both structure and function throughout life and in response to experience. Just as individual differences contribute to variability observed in brain structure and function (see Gu and Kanai, 2014, for a review), mechanisms of neuroplasticity also show significant variability across individuals. Indeed, a growing number of recent studies suggest that the rules and mechanisms that govern cortical plasticity are more variable than previously thought. The purpose of this article is to shed light on the various factors that contribute to neuroplastic variability observed within cortical sensory systems, with a special focus on the auditory system as a model. We will establish the role played by critical periods, plasticity inhibitors, and neuromodulator systems and highlight how these factors interact with other elements such as age, sex, and sensory experience to produce a broad variability of plastic processes. We propose that developing a more robust comprehension of the individual differences that exist within neuroplastic mechanisms can have a significant impact on how clinicians and researchers approach a wide range of neurological and neurodevelopmental disorders. The first section of this paper will introduce the concepts of experience dependent plasticity, critical periods, and plasticity inhibitors. The second portion will provide evidence of how the quality and quantity of sensory inputs reaching the brain influence the rules of plasticity within cortical sensory areas. The third part will illustrate how individual differences in neuromodulator tone can differentially affect brain plasticity within sensory cortices throughout the lifetime.

## Experience-Dependent Plasticity in the Developing and Mature Brain

### Critical Periods for Experience-Dependent Plasticity

Age is a key determinant of experience-dependent cortical plasticity. Important structural and functional changes tend to predominantly occur early in life during time-limited epochs of stimulus-driven plasticity known as *critical periods* (Knudsen, 2004). A well-known example of this limited time-window was provided by the classic monocular visual deprivation studies of Wiesel and Hubel (1963). CPs have since been described in all major sensory systems and in a variety of animal species and their identification has been instrumental in the discovery of the cortical machinery involved in their regulation (see Hensch, 2005 for a review). Many studies of CP plasticity have focused on the rat primary auditory cortex (A1) model, which displays a succession of partially overlapping CPs for various stimulus parameters during development (de Villers-Sidani and Merzenich, 2011). For example, frequency tuning has the earliest and shortest CP in the auditory system (around days 11–14 of life), whereas CPs for more complex sound representations, such as frequency modulation tuning, tend to occur slightly later during early infancy (around days 25–33) (Insanally et al., 2009). Several sensitive periods have also been identified in humans, particularly as they relate to hearing restoration following prelingual deafness and language acquisition. Current evidence suggests that the optimal time for cochlear implantation is before 4 years of life and that implantations performed after 7 years are unlikely to produce satisfactory results (see Kral and Sharma, 2012). Although typically associated with early developmental stages, there is a growing body of evidence demonstrating that CPs can be reopened later in life due to a variety of factors that are still being uncovered. These include damage to peripheral sensory organs (Chino et al., 1992; Diamond et al., 1993; Van Brussel et al., 2011) and changes in the sensory environment (He et al., 2006; Zhou et al., 2011). Recent work has shown that plastic changes in auditory cortex that normally occur within early CPs can even be observed in aging humans and rodents (de Villers-Sidani et al., 2010; Mishra et al., 2014). This suggests that the elements that regulate plasticity change throughout the lifespan and do not only operate around developmental CPs.

### Plasticity Inhibitors and Cellular Brakes

**FIGURE 1:**
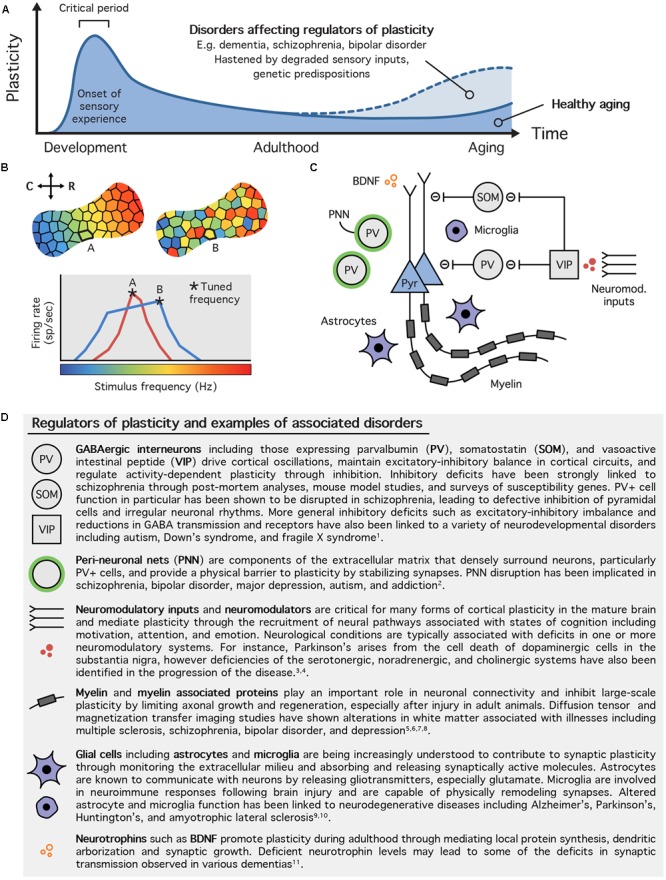
Regulation of experience dependent plasticity. **(A)** Trajectory of experience dependent plasticity during the lifetime. The onset of sensory experience triggers the opening of critical period windows during which the sensory cortex is rapidly organized in response to passive stimulation from the external environment. With maturation, the critical period closes and sensory representations are stabilized. Plasticity continues to take place during adulthood but is tightly regulated by a variety of cellular and molecular processes. These mechanisms tend to decrease with age allowing for non-specific passive experience to elicit plasticity during aging. Disorders that affect regulators of plasticity increase the likelihood for maladaptive plastic changes to take place in the brain. **(B)** Auditory tonotopic map plasticity. Example of a mature tonotopic map from the rat primary auditory cortex (top left) and that of a rat demonstrating irregular plasticity (top right). The tonotopic map typically exhibits a smooth gradient with neurons in the most caudal (C) part of the cortex firing preferentially (or tuned to) low frequencies and neurons in the most rostral (R) part tuned to high frequencies. In unusual plastic states, such as aging and after long-term exposure to white noise, this functional gradient becomes disrupted as tuning of individual neurons becomes less selective (bottom). For example, a neuron’s tuning may shift from being narrow and selective (site A – red line) to broad and flat peaked (site B – blue line), sometimes altering its tuning frequency (**A,B** based on Cisneros-Franco et al., unpublished and Thomas et al., unpublished). **(C)** Some of the major regulators of plasticity in the auditory cortex. Plasticity regulators limit plasticity in the mature brain by controlling the activity of excitatory cells, primarily pyramidal (Pyr) neurons. They include cells such as inhibitory interneurons and glia, structural molecules like peri-neuronal nets and myelin associated proteins, neuromodulatory control from other brain regions, and neurotrophic factors. **(D)** Table of regulators of plasticity and associated disorders. Altered neuroplasticity has been implicated in a variety of neurological disorders including psychiatric, neurodevelopmental, and neurodegenerative disorders. This table highlights just a few examples of disorders associated with specific regulators of plasticity. References: (1) Marín, 2012 (Review); (2) Sorg et al., 2016; (3) Brichta et al., 2013 (Review); (4) Higley and Picciotto, 2014; (5) Wen et al., 2014; (6) Bellani et al., 2016; (7) Mighdoll et al., 2015; (8) Mandl et al., 2015; (9) Singh and Abraham, 2017 (Review); (10) Morris et al., 2013; (11) Arancio and Chao, 2007.

With CP closure, sensory representations are stabilized (Rice and Van der Loos, 1977; Fagiolini et al., 1994; Zhang et al., 2002; de Villers-Sidani et al., 2007). This process requires the maturation of inhibitory (GABAergic) cellular networks and the maintenance of sufficient GABAergic tone in the cortex (Hensch, 2005; Fritschy and Panzanelli, 2014). Any further modification of these networks and associated cortical plasticity is regulated by a series of plasticity inhibitors and molecular brakes, so-called because of their role in limiting plasticity in the mature brain (see Hensch, 2005; Bavelier et al., 2010, for reviews). Functional and structural elements that promote and constrain plasticity include the inhibitory activity of GABAergic interneurons such as parvalbumin positive (PV+) cells (Kuhlman et al., 2013), extracellular matrix components including perineuronal nets (PNNs) (Wang and Fawcett, 2012), and myelin associated proteins (McGee et al., 2005). For a summary of these elements, see **Figure 1**.

Throughout life, the proportion of GABAergic interneurons in the cortex remains relatively stable. However, the number of PV+ and somatostatin positive (SOM+) interneurons decreases with age, indicating that different interneuron subtypes are differentially affected by aging (Stanley et al., 2012; Ouellet and de Villers-Sidani, 2014). Furthermore, PV staining intensity has been shown to be positively correlated with the degree of experience-dependent plasticity (de Villers-Sidani et al., 2008; Zhou et al., 2011). Adult brain CP-like plastic remodeling can be induced by down-regulating cortical inhibition (Fagiolini and Hensch, 2000) or disrupting PNNs (Pizzorusso et al., 2002; McRae et al., 2007; Wang and Fawcett, 2012) or myelin (Kartje et al., 1999; McGee et al., 2005), which form structural barriers to limit plasticity and stabilize cortical representations.

Loss of inhibition during aging could lead to a state of cortical instability where sensory representations are easily distorted by non-specific passive experiences as is the case during CPs (Zhou et al., 2011) (**Figures 1A,B**). Indeed, we recently observed that experience-dependent plasticity is not only paradoxically enhanced, it is also unstable (i.e., producing plastic changes that decayed rapidly in time) in old rats compared to young controls, and was paralleled by a reduction in PV+ cell density, GABA concentration, and PNNs (Cisneros-Franco et al., unpublished). We also found that passive distortions of the auditory map decayed rapidly, indicating an ongoing instability of A1 tuning in the aging cortex. These observations led us to propose that the inhibitory regulation of plasticity, rather than plasticity *per se*, is reduced in the aged brain. This finding has important repercussions for the development of rehabilitation strategies targeted toward aging and opposes the traditional view that aging is a period of limited plasticity.

## Sensory Inputs Reaching the Brain Influence the Rules of Plasticity

### The Quality and Quantity of Sensory Inputs Affect the Timing of Critical Period Windows

Studies of CPs have demonstrated the importance of sensory experience for normal neurodevelopment and sensory map acquisition. The quality and quantity of sensory experience, however, can have diverse effects on CP duration and outcome. Enriched sensory environments, for example, can prolong CP plasticity (Greifzu et al., 2014), stimulate dendritic growth (Leggio et al., 2005; Bose et al., 2010), and improve neuronal response properties (Engineer et al., 2004; Feldman, 2005), whereas deprived or unstructured noisy environments postpone CP onset and maintain cortical neurons in an immature state (Cynader and Mitchell, 1980; Mower, 1991; Fagiolini et al., 1994). In general, the excess presence of a specific stimulus during the CP appears to result in its exaggerated incorporation into the sensory map. For instance, altering the visual environment of the kitten through striped surroundings (Sengpiel et al., 1999) or goggles (Tanaka et al., 2009) shifts the orientation selectivity of visual cortical neurons to prefer the dominant orientation of their environment. In auditory cortex, pure tone pips of a chosen frequency played continuously result in the overrepresentation of that frequency within the tonotopic map (Zhang et al., 2001; de Villers-Sidani et al., 2007). However, there is evidence for hardwired preferences for ethologically relevant stimuli such as tone pips played at a temporal modulation rate similar to that of communication (Kim and Bao, 2009) and vocalizations from members of the same species (Soha and Marler, 2001). The quantity of salient stimuli present during development can also affect the timing of CP closure. Exposure to temporally modulated white noise produces a shorter than usual CP for spectral tuning in auditory cortex, whereas the masking of normal auditory inputs with continuous white noise keeps it open indefinitely (Chang and Merzenich, 2003). Similarly, exposure to bandlimited noise results only in the selective functional and inhibitory maturation of sectors of the tonotopic map outside of the noise band (de Villers-Sidani et al., 2008).

### Sensory Inputs with Low Signal-to-Noise Ratio Can Trigger Plasticity in the Mature Cortex

While the fidelity of sensory inputs has long been known to affect perceptual development, the potential effects of weak, absent, or noisy sensory inputs on mature brain function are only beginning to be understood. Sensory information reaching the brain can be degraded due to exogenous or endogenous factors. Exogenous factors are environmental noise that reduce the discriminability of a stimulus, such as listening to a voice in a crowded room, whereas endogenous factors refer to limits of the peripheral sensory organs or central processing disorders that affect the perception of sensory inputs. In all cases, plastic processes determine how the brain responds and adapts to these challenging perceptual situations and a major goal of neuroscience research should be to understand and integrate our knowledge of these different contexts. We previously demonstrated the similarity between auditory impairments that arise with natural aging in old rats and young adult rats exposed to continuous white noise for 8 weeks (Kamal et al., 2013). Aged rats displayed poor tuning selectivity, neuronal desynchronization, and reduced sensitivity to low-probability sounds, which was nearly indistinguishable from the young adult rats that had been housed in a noisy auditory environment. Furthermore, these impairments were associated with reduced inhibitory interneuron expression and decreased cortical myelin density in both groups of animals. More recently, we observed that exposure to amplitude-modulated noise did not produce the same plastic changes as continuous noise in young adult rats (Thomas et al., unpublished). We concluded that auditory inputs with a high temporal signal-to-noise ratio are protective for auditory function well into adulthood. Together, these findings strongly suggest that noisy sensory inputs, whether originating from the environment or endogenous processes associated with aging could manifest similar functional and structural deficits. Other studies have also demonstrated the ability of noisy environments to induce plastic changes in the mature auditory cortex resulting in impaired function (Pienkowski and Eggermont, 2010; Zheng, 2012; Gourévitch et al., 2014). When returned to normal environments, however, most of these changes appear to be completely or partially reversible indicating ongoing mechanisms of homeostatic plasticity. This in turn suggests that interventions that target plasticity such as enriched environments (e.g., musical training and experience) and discrimination training could be used to counteract or prevent the effects of degraded sensory inputs on mature brains (White-Schwoch et al., 2013; Alain et al., 2014; Mishra et al., 2014).

## Brain Plasticity is Mediated by Neuromodulator Systems

### Neuromodulator Systems As Drivers of Plasticity

In addition to sensory experience, various neuromodulator systems can affect both CPs and adult cortical plasticity by increasing neuronal excitability, improving signal to noise ratio, and controlling the propagation of activity through the cortex (Kirkwood, 2007). Early studies indicated that norepinephrine, a key neurotransmitter of the noradrenergic system, is necessary for ocular dominance column plasticity during the critical period (Kasamatsu and Pettigrew, 1976; Kasamatsu et al., 1979). Subsequent work, however, suggested that both noradrenergic and cholinergic networks need to be impaired to affect cortical plasticity, suggesting a functional redundancy between the two systems (Bear and Daniels, 1983; Bear and Singer, 1986). More recent studies have demonstrated that the cholinergic system is a potent neuromodulator of attention, learning and memory, in both humans (Rokem and Silver, 2010; Beer et al., 2013; Moran et al., 2013; Chamoun et al., 2017) and animal models (Herrero et al., 2008; Hasselmo and Sarter, 2011). Furthermore, Shepard et al. (2015) has provided evidence that mice lacking norepinephrine failed to reorganize auditory cortex frequency representation in response to prolonged sound exposure, suggesting that norepinephrine is a necessary driver of CP plasticity within auditory cortex. The dopaminergic and the noradrenalinergic systems have also been shown to significantly modulate and shape cortical plasticity. For instance, dopamine upregulation has been linked with increases in the auditory cortical representation of paired tones (Bao et al., 2001) and increases in noradrenaline have been shown to increase the threshold of acoustic excitatory responses in auditory neurons (Manunta and Edeline, 1998).

Taken together, these findings highlight the critical role of neuromodulator systems as the main gating mechanisms of plasticity in adult sensory cortex, as well as their important role in shaping cortical function and cognitive abilities. Indeed, both neurochemically boosting cholinergic transmission (Greuel et al., 1988; Voss et al., 2016) and stimulating the basal forebrain — from which the cholinergic neurons project to the cortex — (Kilgard and Merzenich, 1998; Froemke et al., 2007; Kang and Vaucher, 2009; Kang et al., 2014) have been shown to have a significant effect on learning rates and the cortical processing of stimuli. Stimulating the dopaminergic system has also been shown to improve cortical signal-to-noise ratio (Winterer and Weinberger, 2004; Kroener et al., 2009), to enhance visual perceptual performance (Müller et al., 1998; Noudoost and Moore, 2011) and to modulate plasticity within sensory cortex (Bao et al., 2001; Hui et al., 2009). These data provide interesting research avenues worth exploring to develop methods to promote neuroplasticity in situations of learning difficulties or of recovery following brain injury.

### Inter-individual Variability of Neuromodulator Tone Affects Brain Plasticity and Cognition

One of the hallmarks of cognitive processes is the inter-individual variability that exists among healthy individuals. Indeed, a growing body of evidence suggests that this variability is intrinsically linked to variability within the neuromodulator systems. In particular, the potency of the dopaminergic and cholinergic systems changes across the lifespan and cognitive abilities tend to correlate with the maturation of these systems. For instance, the inverted u-shaped function of dopamine signaling (Arnsten, 1998; Goldman-Rakic et al., 2000), where an optimal dopamine level results in improved neuronal function while both insufficient or excessive dopamine levels impair function, is well-suited to model the link between changes in cognitive performance across the lifespan and age-related changes in dopamine signaling, both which also follow an inverted u-shaped function (Störmer et al., 2012). Similarly, cholinergic functions have also been shown to decline during the course of healthy aging (Gibson et al., 1981; McGeer et al., 1984; Voss et al., 2016) and are linked to age-related cognitive and perceptual decline (Everitt and Robbins, 1997; Schliebs and Arendt, 2011). The degeneration of neuromodulatory function with normal aging is likely to contribute to both the diminished and enhanced plasticity observed in aging individuals because neuromodulatory control is weakened overall. While older adults have poorer learning outcomes traditionally perceived as a reduction in plasticity, they are also more vulnerable to maladaptive plastic changes (Mahncke et al., 2006; Oberman and Pascual-Leone, 2013).

There is also an increasing number of studies demonstrating important sex differences regarding neuromodulator levels and how they affect cognition. Research with both animal models and humans have reported that nicotine — a receptor agonist of the cholinergic system—, for instance, can increase learning rates in a sexually dimorphic manner (Levin et al., 1993; Algan et al., 1997). Similarly, animal studies have found that dopaminergic neurotransmission is modulated by sex steroids (Becker, 1990; Booze et al., 1999). In particular, estrogen considerably enhances striatal dopamine synthesis, baseline dopamine release, and the behavioral and neurochemical response to d-amphetamines (Becker, 1990, 1999). It is generally agreed that estrogen has an overall facilitating effect on dopaminergic neurotransmission (Jacobs and D’Esposito, 2011; Uban et al., 2012) and that interactions between estrogen and dopamine significantly affect memory functions (Jacobs and D’Esposito, 2011; Quinlan et al., 2013).

Finally, psychological traits have also been shown to covary with neuromodulator levels. Previous research has identified a relatively strong relationship between dopamine receptors and individual differences in self-reported novelty-seeking personality (Norbury and Husain, 2015) and individual differences in sensation-seeking behaviors have been linked to brain dopamine function (Hamidovic et al., 2009; Derringer et al., 2010). Furthermore, risk-taking behavior in humans can be directly manipulated with dopaminergic drugs, but the effectiveness of such a manipulation depends on baseline sensation-seeking traits (Norbury et al., 2013).

Taken together, these findings provide multiple lines of evidence demonstrating that important inter-individual differences exist within the various neuromodulator systems, and that, therefore, these individual differences are likely also reflected, to a certain extent, in measures of cortical plasticity. Future studies would benefit from taking these individual differences into account when investigating the relationship between neuromodulator tone and cortical plasticity.

### Neuropathological Condition and Drug Treatments Can Alter Neuromodulator Balance

Several neuropathological conditions present with significant neuromodulator imbalances. For instance, hallmark pathophysiological features of schizophrenia and Parkinson’s disease include the disruption of dopaminergic networks, whereas Alzheimer’s disease and multiple forms of dementia are associated with disturbances in the cholinergic and noradrenergic systems. These neuropathological conditions are also often associated with perceptual impairments, which could be caused or exacerbated by neuromodulatory imbalances. In schizophrenia, for instance, in addition to deficits in higher-order processes, deficits can be found throughout the cortex at the level of early sensory processing (Javitt, 2015; Javitt and Freedman, 2015). In particular, schizophrenia has been recently associated with a variety of low-level auditory dysfunctions evidenced by behavioral, electrophysiological, and structural metrics (Javitt and Sweet, 2015), and although more limited, there is also evidence that visual cortex plasticity may also be compromised in the disease (Cavus et al., 2012). Central auditory dysfunction, in the absence of severe peripheral hearing loss, is also associated with high incidences of cognitive decline and Alzheimer’s disease (Gates et al., 2008; Albers et al., 2015). In transgenic mice overexpressing amyloid precursor protein, the presence of Alzheimer’s disease pathology is associated with loss of GABAergic inhibition (Huang and Mucke, 2012) and significant changes in auditory evoked responses within the primary auditory cortex (Wang et al., 2003).

**FIGURE 2:**
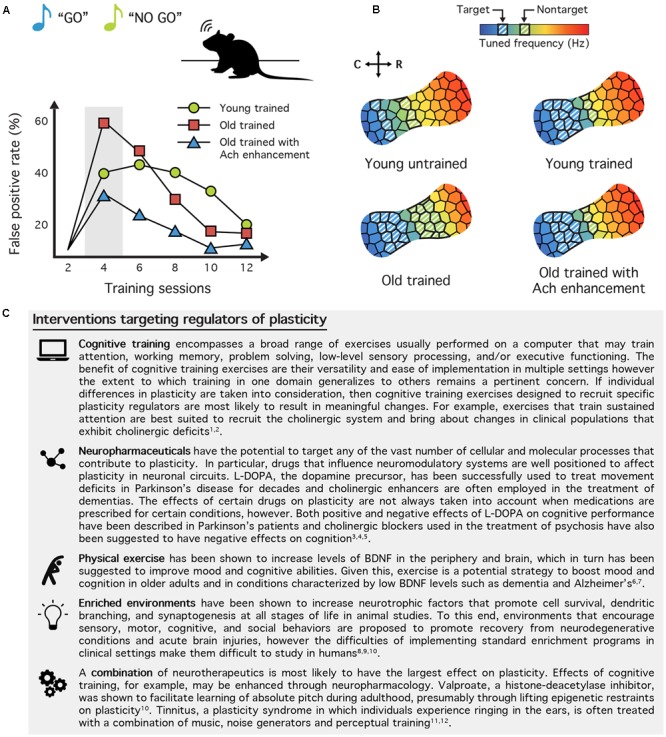
Neurotherapeutic interventions targeting regulators of plasticity. **(A)** Cholinergic enhancement paired with training reduces the probability of false positives (FP) in aged rats. Young adult and old (>24-month-old) rats were trained on a “Go (target frequency)/No-Go (non-target frequency)” auditory perceptual learning task. The FP rate can be used as an indicator of distractibility, and aged humans and rodents tend to have particularly high FP rates during early stages of training. When aged rats were given the cholinesterase inhibitor rivastigmine before each training session their FP rate was halved. This suggests that boosting the cholinergic system can enhance perception and behavioral performance in the elderly by reducing distractibility. **(B)** The tonotopic map of trained aged rats resembles young rats when training is paired with cholinergic enhancement. Compared to a naive, young adult rat (top left), the map of a trained rat (top right) will have a greater proportion of sites tuned to the target stimulus frequency and a smaller proportion tuned to the non-target frequency. This differential representation is believed to help the rat assign more importance to the target tone and ignore the non-target. In old rats (bottom left), however, training results in an equal enlargement of both the target and non-target frequency regions. While these rats are capable of learning the discrimination task, this alternative learning mechanism may result in their elevated FP rate. Indeed, old rats trained with rivastigmine (bottom right), which exhibit less distractibility exhibit map plasticity like young adult rats (**A,B** modified from Voss et al., 2016). **(C)** Table of neurotherapeutic interventions aimed at mechanisms of plasticity. By targeting the various regulators of plasticity, the goal of neurotherapeutics is to utilize the brain’s innate capacity to change to improve learning, memory, and recovery from neurological injury or disease. This brief selection demonstrates a broad range of behavioral, pharmaceutical, and environmental interventions either currently available or under exploration today. References: (1) Vinogradov et al., 2012; (2) Cramer et al., 2011; (3) Cools, 2006; (4) Suraweera et al., 2015; (5) Grinevich et al., 2009; (6) Coelho et al., 2013; (7) Szuhany et al., 2015; (8) Mora et al., 2007; (9) Nithianantharajah and Hannan, 2006; (10) Alwis and Rajan, 2014; (11) Gervain et al., 2013; (12) Zenner et al., 2017.

Several medications have been developed to specifically target the neuromodulator systems involved in neuropathological disorders, and therefore, also affect brain plasticity and sensory processes. In theory, these drugs could be used to target mechanisms of sensory plasticity in healthy adults and be paired with training to ward off perceptual deficits associated with natural aging. For instance, cholinergic antagonists have been shown to significantly improve occipital cortical responsiveness in rats (Kang et al., 2015) and visual perceptual learning in humans (Rokem and Silver, 2010), and recent evidence suggests that these learning effects can last several months after ceasing cholinergic enhancement (Rokem and Silver, 2013). Cholinergic function can also be enhanced through the use of cholinesterase inhibitors such as rivastigmine or donepezil (Colović et al., 2013), which are currently used to treat Alzheimer’s disease and diverse dementia (Ellis, 2005; Birks, 2006). We recently showed in the aged rat that a daily administration of rivastigmine paired with training on an auditory discrimination task led to profound plastic changes within auditory cortex compared with age-matched controls who only underwent perceptual training (Voss et al., 2016) (**Figures 2A,B**). Not only did boosting cholinergic function produce robust frequency map and tuning bandwidth changes within auditory cortex, it also significantly improved the speed with which rats learned to perform the task. Furthermore, the magnitude of the functional changes was found to correlate with each rat’s individual discrimination performance. These results demonstrate that combining perceptual training with neuromodulation of the cholinergic system can restore cortical functional deficits observed as a result of normal aging. Taken together, these findings highlight the therapeutic potential and the powerful potentiating effect of neuromodulator systems for improving the recovery or prevention of age-related and disease-related deficits. See **Figure 2C** for a selection of current and proposed therapeutic interventions targeting various modulators of plasticity.

## Future Directions

The research described here demonstrates that the propensity for experience dependent plasticity throughout life can be more or less potentiated by diverse factors including individual genetic, cellular, molecular, and environmental differences. These findings have lead us to understand that the rules that regulate plasticity are not only more intrinsically variable than were previously thought, but can also be shaped in mature brains. Although plasticity within sensory systems is greatest during time-limited epochs during early development, plasticity regulators in the adult brain can be manipulated by acting on various neuromodulators and by precisely regulating sensory input. Indeed, our lab and others have shown that degraded sensory inputs can trigger plasticity within primary sensory cortex, possibly the result of an adaptive mechanism to facilitate cortical rewiring in cases of neurological injury or trauma to peripheral sensory systems. The idea that plasticity mechanisms can operate throughout the lifespan suggests that many functional properties of sensory neurons can be altered, and even reversed in some cases. This is particularly relevant for neurodegenerative and neuropsychiatric conditions where plasticity mechanisms appear to be dysregulated. Additional research will be required to more completely model age and disease-related plastic changes within sensory cortex, which will then allow us to better tailor stimulus-exposure or behavioral training paradigms to produce the desired functional and behavioral outcome measures. Indeed, without properly establishing a link with behavior, the nature of the reorganization, whether adaptive or maladaptive, will remain difficult to establish. Studies focusing on this goal will be important, with procedures allowing functional perturbation of particular relevance to establish causality.

Moving forward, it will also be necessary to take into account individual differences including age, sex, drug use, and pathological conditions in order to advance personalized treatments that aid learning, memory, and recovery from brain injury and disease. Neuromodulator systems, in particular, display immense variability between individuals. This is particularly evident when considering the huge range of interindividual variability in the effects of cholinergic, dopaminergic and noradrenergic drugs, regardless of the desired outcome measure (Keers and Aitchison, 2010; Tang et al., 2014; Turner et al., 2015). Individual differences in baseline perceptual abilities (Wong and Perrachione, 2007; Perrachione et al., 2011) and in brain connectivity (Lee et al., 2014; Voss and Zatorre, 2015) are also likely to affect learning and recovery rates. As with many medical and health-related fields where personalized and precision medicine are increasingly becoming mainstream, neurotherapeutic interventions targeting mechanisms of plasticity and cognition should also follow an individualized approach by harnessing individual differences to best utilize the brain’s innate capacity to change.

## Author Contributions

PV, MT, JC-F, and EdV-S conceived and wrote the manuscript. MT conceived and designed the figures.

## Conflict of Interest Statement

The authors declare that the research was conducted in the absence of any commercial or financial relationships that could be construed as a potential conflict of interest.
